# Between Bitterness and Sweetness: How Decaffeination and Sweeteners Shape the Sensory Experience of Espresso Coffee

**DOI:** 10.1111/1750-3841.71157

**Published:** 2026-06-18

**Authors:** Kalinca Vitoria Cardoso Cusielo, Alessandra Cazelatto de Medeiros, Erika Yumi Hiramatsu, Helena Maria André Bolini

**Affiliations:** ^1^ Department of Food Science and Nutrition, School of Food Engineering University of Campinas Campinas Brazil

**Keywords:** acceptance, decaffeinated, espresso coffee, external preference mapping, QDA, sensory analysis, stevia, sucralose

## Abstract

The increasing demand for healthier beverages has encouraged the reduction of caffeine and sugar in coffee‐based products. This study evaluated the sensory descriptive profile and consumer acceptance of traditional and decaffeinated espresso coffees sweetened with sucrose, sucralose, and stevia with different rebaudioside A concentrations. The sensory characterization was performed using Quantitative Descriptive Analysis (QDA) with eighteen trained assessors, and acceptance was evaluated by 120 consumers. The physicochemical analyses indicated that decaffeinated coffees showed lower pH values and higher titratable acidity compared to traditional ones. In the sensory evaluation, traditional espressos presented higher scores for brown coffee color, crema creaminess, roasted aroma, and bitterness, while decaffeinated samples exhibited higher red hue (a*) values and more pronounced acidity. Sweeteners influenced multiple sensory attributes: sucrose was associated with higher perceived sweetness and body, while stevia increased perceived acidity, roasted flavor, and sweetness aftertaste. Consumer preference mapping associated higher liking with samples sweetened with sucrose and sucralose. These findings suggest that both the coffee type and sweetening agent significantly affect the sensory perception of espresso coffee, emphasizing the need for formulation strategies that preserve desirable sensory attributes in sugar‐reduced or decaffeinated versions.

**Practical Applications:**

The conclusions reached represent a relevant source of knowledge for the coffee industry in the development and production of coffee‐based beverages. The findings contribute to a better understanding of the behavior and sensory characteristics of sweeteners, offering insights into the perception of sweet and bitter tastes and the factors that influence them, as well as into the sensory profile of the samples, considering that the performance of sweeteners varies according to the product in which they are incorporated.

**Abbreviations**
AAR: acidic aroma
ANOVA: analysis of variance
AST: astringency
ATA: acid taste
BAT: bitterness aftertaste
BCC: brown coffee color
BDY: body
BTA: bitter taste
CAR: coffee aroma
CCS: crema creaminess
CFL: coffee flavor
CRC: brown crema color
CVL: crema volume
DS60: decaffeinated espresso coffee with stevia with 60% rebaudioside A
DS78: decaffeinated espresso coffee with stevia with 78% rebaudioside A
DS97: decaffeinated espresso coffee with stevia with 97% rebaudioside A
DSUC: decaffeinated espresso coffee with sucralose
DSUG: decaffeinated espresso coffee with sucrose
FEA: Faculty of Food Engineering
FFL: fruity flavor
Ovi: overall impression
PCA: principal components analysis
PLS: partial least squares
QDA: Quantitative Descriptive Analysis
RAR: roasted aroma
RFL: roasted flavor
SAR: sweet aroma
SAT: sweetness aftertaste
STA: sweet taste
TS60: traditional espresso coffee with stevia with 60% rebaudioside A
TS78: traditional espresso coffee with stevia with 78% rebaudioside A
TS97: traditional espresso coffee with stevia with 97% rebaudioside A
TSUC: traditional espresso coffee with sucralose
TSUG: traditional espresso coffee with sucrose
VIS: viscosity

## 1 Introduction

Coffee is one of the most consumed beverages worldwide, appreciated for its complex aroma, flavor, and stimulating properties. In recent years, growing health concerns have increased demand for beverages with reduced caffeine and sugar content, including coffee products (Brasil 2012; Market Reports World 2025; Future Market Insights 2025). Decaffeinated coffee offers an alternative for caffeine‐sensitive consumers, while noncaloric sweeteners provide sweetness without added calories. However, both caffeine removal and sucrose substitution can alter the sensory perception of coffee, modifying key attributes such as aroma, flavor, and mouthfeel. The interaction between sweeteners and the coffee matrix may also modify the release of volatile compounds and thus alter the perceived aroma and flavor balance (Paravisini and Guichard 2016).

Noncaloric sweeteners such as stevia and sucralose have been widely used as alternatives to sucrose due to their high sweetening power and low or zero caloric contribution. Stevia, derived from the leaves of *Stevia rebaudiana Bertoni*, contains steviol glycosides such as stevioside and rebaudioside A, which are responsible for its intense sweetness (Higginbotham 1983; Cardello et al. 1999a). However, depending on purity and concentration, these compounds may also be associated with bitter and metallic off‐flavors, which can affect sensory acceptance (Carniel Beltrami et al. 2018; Palazzo and Bolini 2014). In contrast, sucralose generally provides a sweetness profile closer to that of sucrose and is often associated with fewer undesirable sensory attributes (Upreti et al. 2012). Both sweeteners are stable under a wide range of temperatures and pH conditions, making them suitable for use in hot beverages such as coffee. Nevertheless, their interaction with the coffee matrix may influence not only sweetness perception but also the overall sensory profile of the beverage.

Several studies have suggested that the decaffeination process affects the chemical composition of coffee beans, influencing the formation of Maillard reaction products and volatile compounds that contribute to the characteristic sensory profile of espresso coffee (Andueza et al. 2003; Vasquez 2024; Zou et al. 2022). Similarly, replacing sucrose with sweeteners such as stevia or sucralose may influence not only sweetness intensity but also the balance between sweetness, bitterness, and acidity, mainly due to off‐flavors and residual bitterness typical of noncaloric sweeteners like stevia (Carniel Beltrami et al. 2018; Upreti et al. 2012; Bertelsen et al. 2020; Palazzo and Bolini 2014).

Understanding these combined effects is essential for the development of high‐quality, sugar‐reduced coffee products that meet consumer expectations. Therefore, this study aimed to characterize and compare the sensory profile of traditional and decaffeinated espresso coffees sweetened with sucrose, sucralose, and stevia with different rebaudioside A concentrations. To achieve this, the study combined detailed sensory characterization using Quantitative Descriptive Analysis (QDA), physicochemical analyses, and external preference mapping to better understand the relationship between sensory profile and consumer preference.

## 2 Materials and Methods

The following ingredients were used: commercial regular coffee, consisting of the same product available in both caffeinated and decaffeinated versions, in order to ensure greater standardization among the samples, sucrose (União special refined, Tarumã, SP, Brazil), stevia with different rebaudioside A contents (97%, 78% and 60%; Clariant S. A., São Paulo, Brazil), and sucralose (Stevia Farma, Maringa, Brazil).

### 2.1 Sample Preparation

Espresso coffee samples were prepared (7 g/50 mL water) and served to the assessors at the Laboratory of Sensory Sciences and Consumer Studies (LCSEC) of Faculty of Food Engineering (FEA) at University of Campinas (Unicamp), using Jura Impresa F‐50 Classic equipment (JURA AG, Switzerland). This equipment possesses one double extractor and operates at 15 bar pump pressure. The equipment was programmed to deliver a 50 mL espresso per extraction, with the option of dispensing either one 50 mL cup or two 25 mL cups simultaneously, depending on the test requirements. Standard settings for aroma and temperature were maintained, and the extraction time was automatically controlled by the equipment, without the possibility of adjustment.

The samples were classified into traditional espresso coffee (T) and decaffeinated espresso coffee (D). They were sweetened with the following sweetening agents: sucrose (SUG), stevia 97% rebA (S97), stevia 78% rebA (S78), stevia 60% rebA (S60), and sucralose (SUC) totalizing 10 samples: TSUG, TS97, TS78, TS60, TSUC, DSUG, DS97, DS78, DS60, and DSUC. The amount of each sweetener was added in solution to the espresso coffee (0.25 mL/25 mL sample) as described in (Cusielo et al. 2019).

### 2.2 Sensory Evaluation

Sensory evaluation was conducted in individual booths under white fluorescent artificial light and controlled temperature (22°C), following the methodology recommended by (ISO 2007; Meilgaard et al. 2015). Each sample (25 mL) was served immediately after preparation, at 58°C–65°C, in disposable Styrofoam cups, coded with random three‐digit numbers, accompanied by room‐temperature water and cracker biscuit (Bauducco, Minas Gerais, Brazil) for palate cleansing. Samples were presented monadically, using a complete balanced block design (Stone et al. 2012; Wakeling and MacFie 1995). All participants received the informed consent form, approved by the Unicamp Research Ethics Committee (CAAE: 80548524.6.0000.5404).

### 2.3 Quantitative Descriptive Analysis

The recruitment and preselection of the assessors was carried out with 32 volunteers who were submitted to Wald's sequential analysis in order to evaluate their sensory acuity, reproducibility, and ability to discriminate differences between samples (Stone et al. 2012), using triangular difference tests with two coffee samples, with a significant difference at 0.1% between them. As a result, 18 participants were selected as potential panelists (9 women and 9 men).

In the Development of Descriptive Terminology, the repertory grid method (Moskowitz 1983) was applied. The samples were presented in pairs to the assessors, who were asked to list the similarities and differences between them regarding appearance, aroma, taste, and texture. After all responses were compiled, a meeting was performed to select the descriptors, removing terms that lacked consensus or were redundant. Definitions for each term were also established, along with minimum and maximum reference standards. The panel reached consensus on 19 descriptors considered most relevant for espresso coffee (Table 1). Training sessions were then conducted to develop sensory memory and equalization among panelists.

**TABLE 1:** Espresso coffee descriptors used for sensory profiling in the Quantitative Descriptive Analysis.

Descriptors	Definitions	References
**Appearance**		
Brown crema color (CRC)	Light brown hue (caramel tone) of the crema, observed without disturbing the coffee.	**Weak**: Peanut candy (Paçoquita, Santa Helena, Ribeirão Preto, Brazil). **Strong**: Diet creamy dulce de leche (Gotas de Leite, São Paulo, Brazil).
Brown coffee color (BCC)	Dark brown hue characteristic of the liquid portion of espresso coffee	**Weak**: 50 mL of filtered Colombian coffee (Matiz, Bogotá, Colombia). **Strong**: Short espresso (7 g coffee/25 mL water).
Crema volume (CVL)	Amount of crema present on the espresso surface, visually assessed.	**Little**: 50 mL espresso after 1 h of preparation. **Much**: 50 mL espresso + 6 g chantilly cream spray ((Fleischmann, São Paulo, Brazil).
Crema creaminess (CCS)	Apparent viscosity of the crema present on the espresso surface, visually assessed.	**Little**: 50 mL espresso after 1 h of preparation. **Much**: 50 mL espresso + 6 g chantilly cream spray.
**Aroma**		
Coffee aroma (CAR)	Aroma associated with the presence and release of volatile compounds when the beverage is prepared.	**Weak**: 50 mL filtered coffee (Canecão, São Paulo, Brazil). **Strong**: 50 mL filtered Colombian coffee.
Roasted aroma (RAR)	Characteristic aroma associated with the roasting process.	**Weak**: 50 mL filtered Colombian coffee. **Strong**: 50 mL filtered coffee Canecão.
Sweet aroma (SAR)	Characteristic sweet‐like aroma.	**None**: Water. **Strong**: Espresso + 6 g sucrose + 14 drops of 0.01% sucralose solution.
Acidic aroma (AAR)	Characteristic aroma of caffeic acid.	**Weak**: 50 mL decaffeinated filtered coffee. **Strong**: 50 mL filtered Colombian coffee.
**Flavor**		
Sweet taste (STA)	Taste perceived by the presence of sweetening agents (sucrose/sweeteners).	**Little**: 100 mL filtered coffee (Melitta, São Paulo, Brazil) + 3 g sucrose. **Much**: 100 mL filtered coffee Melitta + 15 g sucrose.
Bitter taste (BTA)	Characteristic bitter taste of the product.	**Little**: Double espresso (7 g/100 mL). **Much**: Short espresso (14 g/50 mL).
Coffee flavor (CFL)	Typical flavor of espresso coffee.	**Weak**: Double espresso (7 g/100 mL). **Strong**: Canecão coffee prepared as espresso (7 g/50 mL).
Roasted flavor (RFL)	Characteristic flavor related to the degree of coffee bean roasting.	**Weak**: 100 mL filtered coffee Canecão. **Strong**: Short espresso (14 g/50 mL).
Acid taste (ATA)	Characteristic taste of caffeic acid.	**None**: Water. **Much**: 100 mL filtered Colombian coffee.
Sweetness aftertaste (SAT)	Sweet taste that remains for a period after ingestion.	**None**: Water. **Much**: 0.02% sucralose solution.
Bitterness aftertaste (BAT)	Bitter taste that remains for a period after ingestion.	**None**: Water. **Much**: 0.1% stevia RA97 solution.
Fruity flavor (FFL)	Perceived at the finish, representing a flavor that remains in the mouth after tasting the beverage.	**None**: Water. **Much**: 100 mL filtered Colombian coffee.
Astringency (AST)	Sensation experienced as dryness, commonly described as “mouth puckering.”	**Little**: Concentrated cashew juice diluted in water (1:19, v/v) (Qualitá, Brazil). **Much**: Concentrated cashew juice diluted in water (1:9, v/v).
**Texture**		
Viscosity (VIS)	Sensation perceived upon swallowing coffee.	**None**: Water. **Much**: Ready‐to‐drink cappuccino classic (Três Corações, Brazil) diluted in water (1:1, v/v).
Body (BDY)	Sensation of persistence and fullness of the beverage in the mouth.	**None**: Water. **Much**: Ready‐to‐drink cappuccino classic Três Corações.

The Selection of subjects to perform the QDA is one of the stages of the method and was carried out with four samples, which were presented to the assessors for the evaluation of 19 attributes, in order to select candidates based on the discrimination power between samples, repeatability, and agreement among them (Stone 2018). The selected team of 13 subjects (6 women and 7 men) showed a significant *F*_sample_ for *p* < 0.05 and a nonsignificant *F*_repetition_ for *p* > 0.05.

To perform the QDA, the 13 selected assessors evaluated ten espresso coffee samples for all attributes, using a 9‐cm unstructured linear scale anchored at the left extreme as “weak/little/none” and at the right extreme as “strong/much,” in four repetitions with the aid of FIZZ Software, Biosystems version 2.47B (Biosystemes 2009).

### 2.4 Acceptance Test

The overall impression (Ovi) of the samples was evaluated by 120 consumers (60% female and 40% male), aged 18–55, using a 9‐cm unstructured hedonic scale anchored in the terms “*dislike extremely*” on the left, and “*like extremely*” on the right (Stone et al. 2012) with the aid of FIZZ Software, Biosystems version 2.47B (Biosystemes 2009).

### 2.5 Physicochemical Analyses

Physicochemical analyses of the samples were carried out in triplicate. The evaluated parameters included pH, titratable acidity, soluble solids, and color.

Titratable acidity was determined according to the official (AOAC 1997), based on the principle of sample neutralization until the equivalence point with 0.1 N sodium hydroxide (NaOH), corresponding to the phenolphthalein endpoint (pH 8.2). Due to the dark color of the samples, the titration was monitored using a pH meter (MPA‐210, Tecnopon) to ensure accurate detection of the endpoint.

Soluble solids were quantified by refractometry using a bench‐top refractometer (Carl Zeiss—JENA), following the reference methodology (AOAC 1997).

Color parameters of the espresso coffee samples in the CIELAB system (L*, a*, and b*) were measured using a Hunterlab colorimeter, model ColorQuest II. Measurements were performed under standard illuminant D65 with a 10° observer angle and regular transmission (RTRAN). A white reference plate (C6299 Hunter Color Standard) was used for calibration (Konica Minolta 2007).

### 2.6 Statistical Analysis

The QDA data were analyzed by two factors analysis of variance (ANOVA) (panelist and sample) and Tukey's test averages (*p* ≤ 0.05) using SAS software, version 9.4 (SAS Institute Inc. 2013).

The acceptance test was analyzed by univariate ANOVA and Tukey's test averages (*p* ≤ 0.05) using SAS software, version 9.4 (SAS Institute Inc. 2013).

The external preference map was conducted by first using principal components analysis (PCA), based on a correlation matrix with QDA data, and then by relating each consumer to the PCA space by regression analysis. The correlation between the QDA and overall liking data was determined using partial least squares (PLS) regression analysis using XLSTAT software, version 2025.1.3.1431 (Lumivero 2025), considering a 95% confidence level.

Data from the physicochemical analyses were subjected to ANOVA, and mean comparisons were performed by Tukey's test using SAS software, version 9.4 (SAS Institute Inc 2013).

## 3 Results and Discussion

### 3.1 Physicochemical Analyses

#### 3.1.1 Titratable Acidity and pH

The pH values ranged from 5.40 to 5.43 for traditional coffee samples and from 5.24 to 5.28 for decaffeinated ones, showing significant differences among treatments (*p* ≤ 0.05), as shown in Table 2.

**TABLE 2:** Mean values of physicochemical parameters (pH, titratable acidity, soluble solids, and color) of traditional and decaffeinated espresso coffees sweetened with sucrose and sweeteners.

		Titratable	Soluble	Color parameters		
Samples	pH	acidity	solids	L*	a*	b*
TSUG	5.40^a^	8.18^bc^	8.43^a^	24.95^ef^	0.62^f^	1.53^f^
TS97	5.42^a^	7.90^c^	4.42^b^	25.39^bc^	0.85^c^	1.86^cd^
TS78	5.40^a^	8.18^bc^	4.17^b^	25.18^cd^	0.71^e^	1.68^ef^
TS60	5.42^a^	8.20^bc^	4.42^b^	25.43^b^	0.90^b^	1.98^bc^
TSUC	5.43^a^	8.13^bc^	4.32^b^	25.66^a^	1.08^a^	2.18^a^
DSUG	5.24^b^	8.77^abc^	9.25^a^	24.63^g^	0.53^g^	1.21^g^
DS97	5.25^b^	9.52^a^	3.33^b^	24.95^ef^	0.86^c^	1.80^de^
DS78	5.28^b^	9.13^ab^	3.42^b^	24.77^fg^	0.77^d^	1.70^de^
DS60	5.25^b^	8.97^abc^	3.42^b^	24.92^f^	0.92^b^	1.79^de^
DSUC	5.27^b^	8.55^abc^	3.33^b^	25.15^de^	1.09^a^	2.04^ab^
MSD	0.06	1.12	3.44	0.22	0.03	0.17

*Note*: Traditional with sucrose (TSUG); traditional with stevia with 97% rebaudioside A (TS97); traditional with stevia with 78% rebaudioside A (TS78); traditional with stevia with 60% rebaudioside A (TS60); traditional with sucralose (TSUC); decaffeinated with sucrose (DSUG); decaffeinated with stevia with 97% rebaudioside A (DS97); decaffeinated with stevia with 78% rebaudioside A (DS78); decaffeinated with stevia with 60% rebaudioside A (DS60); decaffeinated with sucrose (DSUC). Means in the same column followed by different superscript letters are significantly different (*p* ≤ 0.05) according to Tukey's test.

Abbreviation: MSD: minimum significant difference.

These results suggest that the decaffeination process may have contributed to a slight reduction in pH, making the decaffeinated beverages more acidic compared to the traditional ones. This difference may be associated with chemical alterations during decaffeination, which may affect the composition and release of acids in the beans. Changes in buffering capacity, possibly related to the removal or transformation of alkaline compounds, may also be involved; however, these mechanisms were not directly measured in this study. In addition, post‐decaffeination thermal treatment may oxidize and hydrolyze chlorogenic acids, forming more acidic phenolic compounds (Franca et al. 2015; Andueza et al. 2003).

The lower pH values in decaffeinated samples corroborate studies that associate the decaffeination process with increased chemical and sensory acidity in espresso coffee (Ky et al. 2001). This change may affect the perception of acidity and bitterness, influencing sensory attributes and potentially reducing acceptance among more sensitive consumers.

#### 3.1.2 Soluble Solids

The total soluble solids (°Brix) values ranged from 3.33 to 9.25, showing significant differences among treatments (*p* ≤ 0.05). Espresso coffees sweetened with sucrose exhibited the highest °Brix values, 8.43 for traditional coffee (TSUG) and 9.25 for decaffeinated coffee (DSUG). This difference is consistent with the chemical composition of sucrose, a disaccharide highly soluble in hot water, which directly increases the refractive density of the beverage and, consequently, its °Brix values.

In contrast, samples sweetened with stevia (TS97, TS78, TS60, and their corresponding decaffeinated versions) and sucralose (TSUC, DSUC) showed significantly lower °Brix values (3.33 to 4.42°Brix). This difference may be related to the fact that noncaloric sweeteners are used in very small quantities, sufficient only to reproduce sweetness equivalent to sucrose, but without significantly contributing to the total soluble solids content.

These results are consistent with previous studies that reported a reduction in °Brix in beverages and foods sweetened with noncaloric sweeteners compared to those containing sucrose (Goraya and Bajwa 2016; Khattab et al. 2017; Mah et al. 2024; Salar et al. 2020; Silva et al. 2025).

Furthermore, the small variation observed between traditional and decaffeinated espresso coffees within each type of sweetener suggests that the matrix type (with or without caffeine) may influence soluble solids, with the type of sweetener being the main determining factor for this variable.

#### 3.1.3 Color Parameters

The color parameters (Table 2) showed significant differences (*p* ≤ 0.05) among treatments, indicating that both the type of coffee (traditional or decaffeinated) and the sweetener used influenced the appearance of the beverage. These variations may be associated with differences in colored compounds formed during roasting (e.g., melanoidins) as well as potential optical effects related to sweetener addition, which may influence the perceived intensity and hue of the beverage (Andueza et al. 2003; Park et al. 2024; Zou et al. 2022).

The L* parameter, representing lightness, ranged from 24.63 to 25.66, indicating subtle yet significant differences among samples. Higher L* values correspond to lighter colorations, while lower values indicate darker and more opaque tones. The TSUC sample showed the highest L* value, differing significantly from the others, whereas DSUG exhibited the lowest, reflecting the combined effect of decaffeination and sweetener type on beverage brightness.

The a* component, associated with the red–green axis, ranged between 0.53 and 1.09. In general, decaffeinated coffees presented higher a* values, suggesting a greater contribution of reddish tones, which may be associated with compositional changes after decaffeination, potentially affecting the balance between brown and reddish compounds. Among sucrose‐sweetened samples, traditional coffee exhibited higher a* values, indicating an intensification of warm tones consistent with differences in colored compounds formed during roasting (Franca et al. 2015), while the observed variations may also be influenced by optical effects after sweetener addition

The b* parameter, corresponding to the yellow–blue axis, ranged from 1.21 to 2.18, showing significant differences (*p* ≤ 0.05) among samples. Higher b* values reflect more yellowish and bright colorations, generally associated with greater formation of thermal reaction products (Franca et al. 2015). This trend was more pronounced in traditional coffees, while decaffeinated samples showed reduced color saturation. The sucralose‐sweetened samples (TSUC and DSUC) exhibited the highest b* values, with no significant difference between them, indicating greater intensity of yellowish tones compared to other formulations.

Overall, traditional espresso coffees exhibited higher lightness and predominance of yellowish tones (L*, b*), resulting in a more vivid golden–brown appearance, whereas decaffeinated samples showed higher a* values, associated with reddish and less saturated tones. These visual differences are consistent with the sensory perception observed in the QDA, where lighter and brighter coffees were described as more balanced, while darker samples were perceived as more intense and roasted in flavor (Upreti et al. 2012; Franca et al. 2015; Park et al. 2024; Zou et al. 2022).

#### 3.1.4 Quantitative Descriptive Analysis

Table 3 presents the mean scores assigned by the trained assessors (*n* = 13) for the 19 sensory attributes evaluated in traditional and decaffeinated espresso coffees sweetened with sucrose, sucralose, and stevia containing different concentrations of rebaudioside A.

**TABLE 3:** Attributes’ averages of the descriptive sensory evaluation by the trained panel (*n* = 13 judges).

Descriptors	Samples										
	Traditional espresso coffee					Decaffeinated espresso coffee					
	TSUG	TS97	TS78	TS60	TSUC	DSUG	DS97	DS78	DS60	DSUC	MSD
Brown crema color (CRC)	5.50^ab^	5.41^abc^	5.31^abcd^	5.61^ab^	5.85^a^	4.91^bcde^	4.18^e^	4.90^bcde^	4.53^cde^	4.44^de^	0.94
Brown coffee color (BCC)	5.31^b^	5.43^b^	5.43^b^	5.61^ab^	6.28^a^	5.77^ab^	5.47^b^	5.46^b^	5.16^b^	5.81^ab^	0.79
Crema volume (CVL)	5.44^bc^	5.22^c^	5.62^abc^	5.77^abc^	6.15^ab^	6.36^a^	5.71^abc^	5.91^abc^	5.56^bc^	5.85^abc^	0.79
Crema creaminess (CCS)	6.60^a^	6.27^abcd^	6.39^ab^	6.64^a^	6.34^abc^	5.96^bcd^	5.97^bcd^	5.76^cd^	6.13^abcd^	5.75^d^	0.59
Coffee aroma (CAR)	5.16^a^	4.46^cd^	4.97^abc^	5.13^ab^	4.55^abcd^	4.47^cd^	4.62^abcd^	4.28^d^	4.50^bcd^	4.23^d^	0.64
Roasted aroma (RAR)	5.87^ab^	5.65^ab^	5.98^a^	5.73^ab^	6.05^a^	5.12^bc^	5.39^abc^	5.48^abc^	5.48^abc^	4.65^c^	0.84
Sweet aroma (SAR)	4.60^ab^	4.48^ab^	4.70^a^	4.60^ab^	4.03^abc^	3.42^c^	4.19^abc^	3.83^bc^	4.36^ab^	3.85^abc^	0.86
Acidic aroma (AAR)	2.64^a^	2.15^a^	2.77^a^	2.51^a^	2.58^a^	2.06^a^	2.69^a^	2.48^a^	2.21^a^	2.71^a^	0.72
Sweet taste (STA)	6.27^a^	4.96^bcde^	5.22^bcd^	4.74^cde^	5.65^abc^	5.69^abc^	5.70^ab^	4.14^e^	4.17^e^	4.49^de^	0.95
Bitter taste (BTA)	3.59^d^	5.05^abc^	5.87^a^	5.44^ab^	4.62^bc^	2.37^e^	3.68^d^	4.85^bc^	4.22^cd^	3.48^d^	0.87
Coffee flavor (CFL)	4.68^a^	4.79^a^	4.91^a^	4.88^a^	5.01^a^	4.40^a^	4.74^a^	4.73^a^	4.80^a^	4.38^a^	0.63
Roasted flavor (RFL)	2.96^e^	5.29^a^	4.42^abc^	3.95^bcd^	3.36^de^	2.74^e^	4.82^ab^	4.68^ab^	3.68^cde^	2.95^e^	0.95
Acid taste (ATA)	4.93^bcd^	5.61^ab^	5.88^a^	5.58^abc^	5.50^abc^	3.78^e^	4.77^cd^	4.80^cd^	4.83^cd^	4.36^de^	0.74
Sweetness aftertaste (SAT)	3.63^def^	5.05^ab^	5.87^a^	5.77^a^	4.32^bcd^	2.19^g^	3.28^ef^	4.05^cde^	4.72^bc^	3.16^f^	0.87
Bitterness aftertaste (BAT)	1.82^a^	1.79^a^	1.88^a^	2.15^a^	2.04^a^	2.29^a^	2.35^a^	2.06^a^	1.95^a^	1.95^a^	0.59
Fruity flavor (FFL)	2.05^d^	2.39^bcd^	3.37^a^	2.72^abcd^	2.64^bcd^	2.12^cd^	2.98^ab^	2.80^abc^	2.82^abc^	2.67^abcd^	0.72
Astringency (AST)	2.18^bc^	2.45^ab^	2.74^a^	2.52^ab^	2.70^ab^	1.70^c^	2.55^ab^	2.34^ab^	2.59^ab^	2.18^bc^	0.54
Viscosity (VIS)	3.78^a^	3.28^ab^	3.32^ab^	3.52^ab^	3.63^ab^	3.60^ab^	3.30^ab^	3.08^b^	3.39^ab^	3.28^ab^	0.62
Body (BDY)	3.20^a^	2.66^ab^	2.84^ab^	3.00^ab^	2.78^ab^	2.95^ab^	2.56^b^	2.84^ab^	2.79^ab^	2.61^ab^	0.61

*Note*: Traditional with sucrose (TSUG); Traditional with stevia with 97% rebaudioside A (TS97); traditional with stevia with 78% rebaudioside A (TS78); Traditional with stevia with 60% rebaudioside A (TS60); traditional with sucralose (TSUC); decaffeinated with sucrose (DSUG); decaffeinated with stevia with 97% rebaudioside A (DS97); decaffeinated with stevia with 78% rebaudioside A (DS78); decaffeinated with stevia with 60% rebaudioside A (DS60); Decaffeinated with sucrose (DSUC). Means in the same row followed by different superscript letters are significantly different (*p* ≤ 0.05).

Abbreviation: MSD: minimum significative difference.

The trained panel identified significant differences (*p* ≤ 0.05) among the espresso samples for most of the evaluated attributes, reflecting the combined effect of coffee type and sweetener on the sensory profile of the beverages.

Among the 19 sensory attributes evaluated, no significant differences were observed for three, acidic aroma (AAR), coffee flavor (CFL) and bitterness aftertaste (BAT), indicating similarities between the traditional and decaffeinated espresso coffees, regardless of the sweetening agent.

Regarding appearance attributes, sample TS60 showed the highest mean scores for brown crema color (CRC), brown coffee color (BCC), and crema creaminess (CCS), while sample DSUG presented the highest score for crema volume (CVL). The behavior of attribute BCC followed the instrumental color parameters, with higher scores in traditional samples characterized by golden–brown coloration and lower scores in decaffeinated ones, which showed darker and reddish tones, consistent with the color results.

Among the decaffeinated samples, DSUG, DS97, and DS78 showed higher CVL values, suggesting that even after caffeine removal, the interaction between the decaffeinated matrix and sweeteners may favor crema formation and stability. The sucrose‐sweetened sample (DSUG) and those with stevia containing higher REB A purity (DS97 and DS78) appeared to contribute to better crema structure, possibly by influencing viscosity and surface properties of the beverage, resulting in a perception similar to that observed in traditional coffees, as shown in Table 4. For aromatic attributes, roasted aroma (RAR) showed higher values in traditional samples, indicating that the decaffeination process may be associated with lower aroma intensity, potentially related to changes in volatile compounds (Andueza et al. 2003), which were not directly measured.

**TABLE 4:** *p*‐values from ANOVA for each attribute.

		Effect	
Descriptors	Coffee	Sweetener	Coffee*sweetener
Brown crema color (CRC)	0.0001	0.5782	0.2736
Brown coffee color (BCC)	0.5424	0.0112	0.1416
Crema volume (CVL)	0.0898	0.1322	0.0364
Crema creaminess (CCS)	0.0001	0.223	0.8485
Coffee aroma (CAR)	0.0015	0.2194	0.2058
Roasted aroma (RAR)	0.0001	0.6285	0.1152
Sweet aroma (SAR)	0.0004	0.1391	0.146
Acidic aroma (AAR)	0.3896	0.2723	0.0174
Sweet taste (STA)	0.0013	0.0001	0.002
Bitter taste (BTA)	0.0001	0.0001	0.9737
Coffee flavor (CFL)	0.0262	0.4375	0.4559
Roasted flavor (RFL)	0.2247	0.0001	0.7428
Acid taste (ATA)	0.0001	0.0001	0.8031
Sweetness aftertaste (SAT)	0.0001	0.0001	0.5649
Bitterness aftertaste (BAT)	0.0836	0.9714	0.096
Fruity flavor (FFL)	0.7269	0.0001	0.0403
Astringency (AST)	0.0107	0.0002	0.1029
Viscosity (VIS)	0.1421	0.1037	0.9122
Body (BDY)	0.1946	0.0874	0.9609

^a^Means that the *p*‐value is less than 0.0001.

^b^ *p*‐values ≤ 0.05 indicate significative difference for the sensory attribute.

Sweet taste (STA) was more intense in samples sweetened with sucrose (TSUG and DSUG), followed by DS97 and TSUC, suggesting higher perceived sweetness intensity. Samples with lower‐purity stevia (60%) presented lower perceived sweetness, likely due to the presence of bitter steviol glycosides, which have been associated in the literature with lingering bitter and metallic taste that may influence sweetness perception and consumer acceptance (Carniel Beltrami et al. 2018).

Bitter taste (BTA) was more pronounced in stevia‐sweetened samples, suggesting bitterness enhancement by steviol glycosides, while sucrose‐sweetened samples exhibited lower values, consistent with a masking effect of sucrose on bitter compounds (Bertelsen et al. 2020). Stevia (especially rebaudiosides A–C) may interact with BTA receptors, which may explain the residual bitterness frequently reported by consumers (Choi et al. 2024).

Acid taste (ATA) and fruity flavor (FFL) attributes showed higher scores in sweetener‐containing samples compared to sucrose‐sweetened ones, within each coffee group (traditional and decaffeinated). Sweetness aftertaste (SAT) and roasted flavor (RFL) showed similar behavior, with higher intensities in stevia‐sweetened samples, followed by sucralose and, lastly, sucrose, also within each espresso group.

These results suggest that the type of sweetener influences the relationship between acid–sweet and perceived flavor profile. Noncaloric sweeteners may enhance acidic and fruity notes, which may be associated with changes in aroma perception and its integration with taste, potentially related to the absence of sucrose (Paravisini and Guichard 2016). Moreover, the presence of steviol glycosides may interact with gustatory and aromatic receptors, intensifying acidity and fruity perception and thus modifying the beverage's sensory complexity, as suggested in the literature (Upreti et al. 2012; Bertelsen et al. 2020; Knoop 2011). Although underlying mechanisms were not directly investigated, these interpretations are presented as hypotheses to explain the observed sensory differences.

Astringency (AST) and body (BDY) attributes also showed significant differences. For AST, TSUG exhibited the highest score, while DS78 presented the lowest. For BDY, TSUG showed the highest intensity and DS97 the lowest, indicating that both coffee type and sweetener affect the tactile perception and structural properties of the beverage.

ANOVA showed significant effects (*p* ≤ 0.05) for several sensory attributes, suggesting that coffee type and sweetener act independently and interactively on the sensory profile. Coffee type significantly influenced attributes such as CRC, CCS, coffee aroma, RAR, sweet aroma, STA, BTA, CFL, ATA, SAT, and AST. These results indicate that caffeine removal affects not only taste and flavor but also aromatic and tactile properties, altering the perception of bitterness, acidity, and BDY (Andueza et al. 2003; Franca et al. 2015).

In general, traditional espresso coffees exhibited greater RAR and bitterness intensity, while decaffeinated espresso coffees were characterized by milder notes and perceptibly different sweetness, consistent with previous studies on the sensory impact of decaffeination (Andueza et al. 2003; Franca et al. 2015)

The type of sweetening agent significantly influenced attributes such as BCC, STA, BTA, RFL, ATA, SAT, FFL, and AST, showing that sweeteners affect multiple sensory dimensions. Sucrose tends to promote gustatory balance and higher overall acceptance, while stevia and sucralose, depending on rebaudioside A purity, may intensify bitter, acidic, or astringent notes, altering the beverage's sensory balance (Upreti et al. 2012; Knoop 2011; Azevedo 2013; Palazzo and Bolini 2014; Azevedo et al. 2017).

Significant coffee–sweetener interactions were observed for CVL, AAR, STA, and FFL, indicating that the sensory impact of the sweetener depends on coffee type. In decaffeinated coffee, differences among sweeteners tend to be more perceptible, possibly due to reduced aromatic complexity and bitterness, which increases sensitivity to sweetness and acidity variations (Prada et al. 2025; Choi et al. 2024).

#### 3.1.5 Acceptance Test

In the acceptance test, no significant differences were observed among the samples for appearance, aroma, and texture; however, significant differences were found for flavor and Ovi (*p* < 0.05). Samples sweetened with sucrose and sucralose presented the highest acceptance means, with no significant difference between them, while those sweetened with stevia were the least accepted, which may be related to the bitter and residual taste characteristic of this sweetener (Cusielo et al. 2019). These results are consistent with previous studies reporting higher acceptance of beverages sweetened with sucrose and sucralose compared to those with stevia (Carniel Beltrami et al. 2018; Cardello et al. 1999b, Cardello et al. 2000; Higginbotham 1983; Serbai et al. 2014).

**FIGURE 1:**
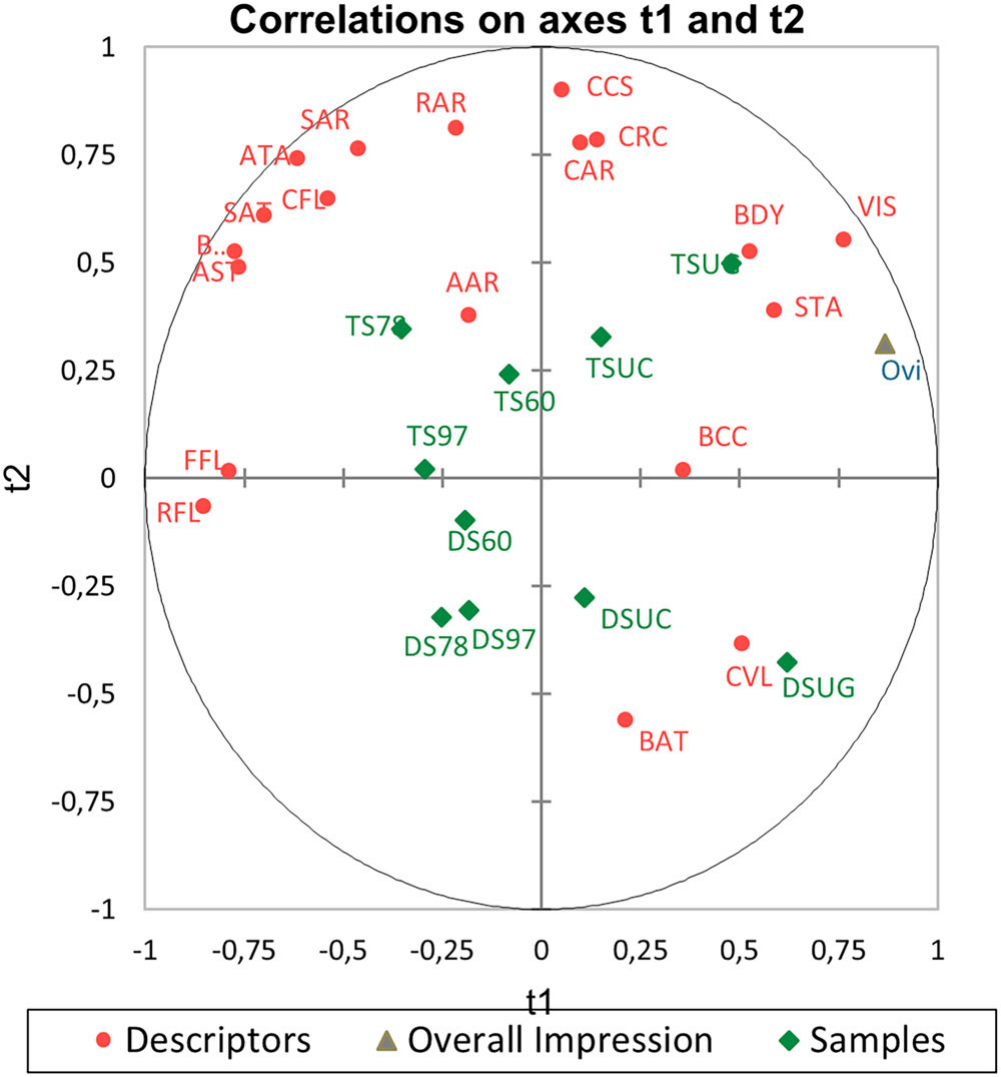
External preference map for the attribute overall impression for traditional and decaffeinated espresso coffees.

The external preference map (Figure 1) allows visualization of the samples (green diamonds) together with the sensory attributes (circles) that characterize them. The sensory attributes represent the descriptors evaluated in the QDA, which contribute to the sensory differentiation among the samples. Principal Components 1 and 2 explain 85% of the total variance. The vectors indicate the correlation of each sensory attribute with the first two principal components, and the samples are positioned according to their sensory and hedonic relationships.

A clear separation was observed among the samples, with well‐defined positions in the quadrants. Traditional espresso coffee samples were located in the upper region of the map, while decaffeinated samples were positioned in the lower region. In addition, samples sweetened with sucrose and sucralose were distributed on the right side, whereas those containing stevia were grouped on the left side, indicating a sensory and hedonic distinction associated with both the coffee type and the sweetener used.

Samples sweetened with sucrose and sucralose (TSUG and TSUC) were positioned in the positive quadrant of both axes and showed positive associations with attributes such as STA, BDY, viscosity, CAR, CRC, CCS, and BCC, being located close to the vector of Ovi. These attributes contributed significantly to consumer acceptance, reinforcing the role of sucrose and sucralose in maintaining the typical and pleasant sensory profile of espresso coffee.

Decaffeinated samples sweetened with stevia (DS97, DS78, and DS60) clustered in the opposite quadrant, near the vector of RFL, which is associated with more intense and bitter sensory notes. This correlation suggests that the residual taste of stevia, combined with RFL perception, negatively affected Ovi. Similar results were reported in previous studies, which identified bitterness and stevia's residual taste as limiting factors for the acceptance of coffee‐based beverages (Cusielo et al. 2019; Palazzo and Bolini 2014; Bertelsen et al. 2020).

The triangle representing Ovi was positioned near traditional samples sweetened with sucrose and sucralose, corroborating the mean acceptance scores. The greater distance of stevia samples from the Ovi reinforces their lower acceptance.

The distribution of samples and attributes shows a clear separation between traditional (TSUG, TSUC, TS78, TS97, TS60) and decaffeinated espresso coffees (DSUG, DSUC, DS78, DS97, DS60), indicating that consumer preference depends not only on the type of sweetener but also on the coffee type. This grouping reveals distinct sensory profiles and confirms that only a small portion of consumers prefer stevia‐sweetened samples.

The results of the external preference map confirm and complement the findings of the acceptance test: samples sweetened with sucrose and sucralose are consistently associated with positive sensory attributes and a favorable Ovi. Stevia‐sweetened samples, on the other hand, are clearly associated with negative attributes, such as bitterness and undesirable residual notes, which contribute to their lower acceptance. These findings highlight the importance of considering the interaction between coffee type, sweetening agent, and individual perception in shaping sensory preferences.

## 4 Conclusion

The results suggest that both the type of coffee (traditional or decaffeinated) and the sweetening agent significantly affected the physicochemical and sensory characteristics of espresso. Decaffeinated samples showed lower pH and higher titratable acidity, suggesting that the decaffeination process may increase the beverage's acidity. Sucrose addition resulted in higher soluble solids and brighter, golden–brown color, while samples sweetened with stevia and sucralose exhibited lower °Brix values and darker hues.

In the sensory analysis, traditional coffees showed higher intensities of RAR, bitterness, and BDY, whereas decaffeinated coffees were perceived as smoother and slightly more acidic. Among sweeteners, sucrose was associated with higher perceived sweetness, while stevia was associated with higher perception of acidity, fruity notes, and bitterness, especially at lower purities of rebaudioside A. Sucralose produced an intermediate sensory profile, close to that of sucrose.

Consumer acceptance confirmed these results: samples sweetened with sucrose and sucralose achieved the highest acceptance scores, whereas stevia‐sweetened beverages were less preferred due to their residual bitterness. Overall, the findings highlight that the type of sweetener may have a stronger impact on the sensory profile and consumer preference than caffeine content, emphasizing the need to optimize sweetener choice in espresso formulations to maintain balance and acceptability.

Overall, this study extends previous research by integrating descriptive sensory analysis, physicochemical data, and consumer preference, providing a more comprehensive understanding of the factors influencing espresso coffee perception.

## Author Contributions

**Kalinca Vitoria Cardoso Cusielo**: methodology, software, data curation, investigation, validation, formal analysis, visualization, project administration, writing – original draft, writing – review and editing, funding acquisition. **Alessandra Cazelatto de Medeiros**: conceptualization, software, methodology, formal analysis, writing – review and editing. **Erika Yumi Hiramatsu**: writing – review and editing, visualization. **Helena Maria André Bolini**: supervision, conceptualization.

## Conflicts of Interest

The authors declare no conflicts of interest.
